# Platelets in preeclampsia: an observational study of indices associated with aspirin nonresponsiveness, activation and transcriptional landscape

**DOI:** 10.1186/s12916-025-04132-9

**Published:** 2025-06-09

**Authors:** Gashaw Garedew Woldeamanuel, Kenean Getaneh Tlaye, Xueqin Wang, Long Nguyen-Hoang, Qiongjie Zhou, Yinan Wang, Bo Wah Leung, Yao Wang, Liona C. Poon, Chi Chiu Wang

**Affiliations:** 1 Department of Obstetrics and Gynaecology, Prince of Wales Hospital, The Chinese University of Hong Kong, Shatin, Hong Kong; 2 Department of Biomedical Sciences, School of Medicine, Wolkite University, Wolkite, Ethiopia; 3 Department of Obstetrics, Obstetrics and Gynecology Hospital of Fudan University, Shanghai, China; 4 Li Ka Shing Institute of Health Sciences, Faculty of Medicine, The Chinese University of Hong Kong, Shatin, Hong Kong

**Keywords:** Platelets, Preeclampsia, Aspirin, Transcriptome

## Abstract

**Background:**

Platelets play critical roles in the pathogenesis of preeclampsia, including thrombosis, endothelial dysfunction and inflammation. However, preeclampsia-associated changes in platelet gene expression and activation at the maternal–foetal interface remain unknown. Moreover, aspirin nonresponsiveness in high-risk pregnancies underscores the need for low-cost biomarkers to identify nonresponders. Nevertheless, the changes of platelet indices in women who develop preeclampsia despite aspirin prophylaxis have not yet been evaluated. In this study, we aimed to investigate the changes in platelet indices associated with aspirin nonresponsiveness, activation state and transcriptional landscape in preeclampsia.

**Methods:**

Platelet indices were compared between aspirin-responsive and nonresponsive women. Logistic regression analysis was performed to determine the associations between platelet indices and aspirin nonresponsiveness. Opal immunofluorescence staining was performed to evaluate the expression of platelet-specific (CD42b) and activation (CD62P) markers in placental villous and decidual tissues. RNA sequencing (RNA-seq) was performed to investigate the transcriptomic profile of platelets.

**Results:**

A decrease in platelet count (PC) during the second trimester as well as an increase in mean platelet volume (MPV) and a lower PC/MPV ratio in the third trimester were significantly associated with the subsequent development of aspirin nonresponsiveness. We observed significantly greater expression of CD62P in the placental villous and CD42b in the decidua of the preeclamptic group than in those of the nonpreeclamptic group. Colocalization analysis of CD42b and CD62P revealed that the preeclamptic placenta and decidua presented significant platelet activation. RNA-seq analysis revealed a total of 20, 618 and 1819 transcripts in the peripheral blood, placental villous and decidua of preeclamptic women, respectively. Functional analysis revealed that the PI3K-Akt and Wnt signalling pathways were significantly enriched in the placental villous and decidua of preeclamptic patients, respectively. RT‒qPCR analysis confirmed the upregulation of FKBP5, LAMA5, FZD5 and FGG mRNA expression in preeclampsia.

**Conclusions:**

Our findings suggest that PC in the second trimester and PC, MPV and PC/MPV ratio in the third trimester may be useful for assessing aspirin nonresponsiveness in women at high risk of preeclampsia. Furthermore, our findings demonstrate that preeclampsia is associated with increased platelet activation and significant enrichment of signalling pathways involved in platelet activation.

**Supplementary Information:**

The online version contains supplementary material available at 10.1186/s12916-025-04132-9.

## Background

Preeclampsia is a common pregnancy disorder that can lead to severe complications, including eclampsia, maternal organ damage, preterm birth, growth restriction and intrauterine death [1, 2]. Although the exact mechanisms remain unclear, it is believed to originate from abnormal placental development, resulting in impaired maternal vascular adaptation and endothelial dysfunction. This, in turn, triggers a cyclical cascade of systemic events, including abnormal immune responses, inflammation and oxidative stress [3, 4].

Haematological complications associated with preeclampsia, including disturbances in platelet function, have long been recognised [5, 6]. A growing body of evidence has shown that platelet activation plays an important role in the pathogenesis of preeclampsia, including thrombosis, endothelial dysfunction and inflammation, although the underlying mechanisms remain unclear [7–11]. Several markers of platelet activation, such as P-selectin [12, 13], platelet factor 4 (PF4) [14] and Annexin-V, have been investigated in preeclampsia [15]. A study by Theilen et al. revealed that women with higher levels of circulating PF4 before conception and during early pregnancy are at increased risk for hypertensive disorders of pregnancy [16]. In addition, other studies have demonstrated that platelet activation can occur even before preeclampsia develops [17–19].

A growing need exists to pinpoint inexpensive platelet parameters that may link platelet changes and preeclampsia. We and others have demonstrated that platelet count (PC) is significantly lower in preeclamptic pregnancies than in normotensive pregnancies [20–22]. The mean platelet volume (MPV), a marker of platelet size and activation, has also been found to be increased in preeclamptic women [23, 24]. However, further well-designed longitudinal studies are still needed to evaluate the predictive performance of these parameters for preeclampsia.

Prophylactic low-dose aspirin has long been recognised for its preventive effect on preeclampsia [25]. Platelets are the main target of aspirin, which inhibits the platelet cyclooxygenase-1 (COX-1) enzyme. COX-1 inhibition blocks thromboxane A2 (TXA2) synthesis, leading to the inhibition of TXA2-dependent platelet activation [26, 27]. However, the platelet response to aspirin is variable, so not all patients may benefit equally from aspirin [28–30]. In light of the effects of aspirin, identifying platelet markers that are associated with aspirin nonresponsiveness is important [9]. Platelet indices, including PC, may provide insight into the global platelet response [31]. However, the changes in platelet indices associated with aspirin use in women at high risk of preeclampsia have not been studied.

In addition to numerous molecules stored in platelet granules, platelets contain a variety of RNA species, including messenger RNA (mRNA) and long noncoding RNAs (lncRNAs). An alteration in platelet RNA expression can affect platelet function [32]. The nucleic acid content of platelets can be altered in response to local or systemic conditions [33]. Studies have shown that diseases such as sepsis [34], cancer [35, 36], coronavirus disease 2019 infection [37] and cardiovascular disease [11] can alter the transcriptome of platelets. It is also important to identify platelet transcripts that differ between preeclamptic and healthy pregnancies to better understand the molecular mechanisms of platelet‒preeclampsia interactions.

In human pregnancy, maternal platelets can be sequestered in the placenta and interact with trophoblasts [38, 39]. An exaggerated activation of platelets and the subsequent release of their factors can impact the maternal‒foetal interface environment [17, 40, 41]. Platelets may play a role in abnormal placentation through thrombosis, placental angiogenesis and maternal vascular remodelling, although the exact mechanisms involved remain obscure [16]. Moreover, the release of inflammatory cytokines and chemokines from adhering maternal platelets may lead to an inflammatory environment in the placenta [17, 42]. However, whether the degree of platelet activation is altered in the preeclamptic placenta and decidua remains to be determined.

The connections between preeclampsia and platelets have not been fully explored, even though platelets are critical to its pathogenesis and potential adverse consequences [6–8].To address this, we comprehensively investigated the platelet changes in women with preeclampsia. Initially, we evaluated the temporal changes of platelet indices across gestation in women who subsequently develop preeclampsia despite aspirin prophylaxis. Nonresponsiveness to aspirin in high-risk pregnancies underlines the critical unmet need for low-cost and easily accessible biomarkers to identify nonresponders, allowing alternative intervention strategies and improving maternal-foetal outcomes. Measurement of platelet indices is easy and inexpensive in clinical settings [20, 21]. Since aspirin targets platelets [26, 27], understanding the changes in these parameters could help guide treatment in high risk women for preeclampsia. Next, we assessed the degree of platelet activation at the maternal-foetal interface by measuring the expression levels of platelet and activation markers using Opal immunofluorescence staining. At last, we performed RNA sequencing (RNA-seq) on platelets and further examined the transcriptional dysregulation in platelets during preeclampsia. By elucidating the platelet transcriptome changes in preeclamptic patients, our study reveals insights into the molecular alterations of platelets associated with preeclampsia, providing directions in mechanisms underlying platelet dysregulation in preeclampsia.

## Methods

This study was reported in accordance with the Strengthening the Reporting of Observational Studies in Epidemiology (STROBE) guidelines for observational studies (Additional file 1).

### Study population

This was an ancillary study to the Asian-based stepped wedge cluster randomised trial named Implementation of First-trimester Screening and Prevention of Preeclampsia (FORECAST, ClinicalTrials.gov identifier: NCT03941886). This multicentre trial aims to evaluate the efficacy, acceptability, and safety of first-trimester screening and prevention for preterm preeclampsia. Women who consented to participate in this trial underwent preeclampsia screening based on the Fetal Medicine Foundation (FMF) prediction algorithm, which encompasses a combination of maternal factors and measurements of mean arterial pressure, uterine artery pulsatility index (UtA-PI) and serum placental growth factor (PlGF) at 11–13^+6^ weeks of gestation [43, 44]. The FMF model determines the conditional probability of developing preeclampsia using Bayes theorem, a mathematical formula for estimating patient-specific risks of preeclampsia [44, 45]. Women with a predicted risk of ≥ 1 in 100 are considered to be at high risk for preterm preeclampsia [2, 46]. Low-dose aspirin (i.e., 160 mg/night) was given to high-risk women from < 16 weeks until 36 weeks of gestation or until delivery in the case of early delivery or the onset of preeclampsia before 36 weeks. Compliance with aspirin intake was measured on the basis of the percentage difference between the number of tablets consumed and the number of tablets prescribed. The exclusion criteria for this trial included multiple pregnancies, major foetal defects, and nonviable foetuses. A detailed description of the study design and results of the FORECAST study has been published [47].

### Inclusion and exclusion criteria

The inclusion criteria were women with singleton pregnancies who participated in the FORECAST trial, were screened as having a low or high risk for preterm PE and gave birth to normal live babies. For the platelet indices study, women with pre-existing hematologic comorbidities or coagulation defects, gestational hypertension and those taking other medications were excluded. For the staining and RNA-seq experiments, the exclusion criteria included chronic hypertension, gestational hypertension, autoimmune diseases, diabetes mellitus, preexisting hematologic comorbidities or coagulation defects. Women who currently smoke or take other medications with known effects on platelets were also excluded.

### Definitions

Preeclampsia was defined according to the International Society for the Study of Hypertension in Pregnancy criteria as diastolic blood pressure ≥ 90 mmHg and/or systolic blood pressure ≥ 140 mmHg plus proteinuria or dysfunction of maternal organs or placental insufficiency. Preterm and term preeclampsia were defined as preeclampsia requiring delivery at < 37 and ≥ 37 weeks of pregnancy, respectively [48]. In this study, women who developed preeclampsia despite aspirin treatment were considered nonresponsive, whereas those who did not develop preeclampsia with aspirin treatment were considered responsive [28, 29]. Moreover, the non-preeclamptic women who have not been on aspirin were screened as having a low risk for preeclampsia, a normal singleton pregnancy and no chronic diseases. Preeclamptic women without aspirin were those who did not take aspirin prophylaxis due to low risk of developing preeclampsia, declined to take aspirin or had contraindications to aspirin.

### Platelet indices

Data on platelet indices (i.e., PC and MPV) were obtained from the computerised records of women who gave birth to a live singleton baby at Prince of Wales Hospital, Hong Kong, between July 2020 and December 2023. This study included all women with at least one platelet parameter measured in the current pregnancy. Platelet indices measured at 9–13 (before aspirin administration), 20–24, and 30–34 weeks of gestation were extracted for analysis. In the preeclamptic group, all measurements were made before diagnosis. For complete blood cell count tests, 2–3-ml blood samples were obtained via antecubital venipuncture with ethylenediaminetetraacetic acid (EDTA)-coated vacutainer tubes. Platelet indices were then measured with an automated blood cell counter (Sysmex XN-350 automated analyser) within 2 h of sample collection.

### Human sample collection and processing

Maternal peripheral blood samples were obtained via venipuncture and collected in 10-ml EDTA-coated vacutainer tubes. These samples were obtained before delivery and processed within 6 h. Moreover, placental villous and decidual tissues were collected within 30 min postdelivery. The placenta was placed at the processing station with the maternal surface facing up to dissect the decidual tissue from three randomly selected sites. The placenta was placed with the foetal surface uppermost to collect the placental villous tissue from three randomly selected sites distal to the cord insertion site, and the foetal membrane was trimmed. All the dissected tissue samples were washed thoroughly with cold phosphate-buffered saline (PBS). The tissues were further minced into smaller pieces. A portion of each sample was fixed in a 10% neutral buffered formalin solution (HT501128, Sigma‒Aldrich) for 48 h at room temperature (RT). Following fixation, the samples were processed overnight, including dehydration, clearing and paraffin infiltration, using an automated Excelsior ES tissue processor (Thermo Fisher Scientific). The samples were then embedded in paraffin blocks. Formalin-fixed paraffin-embedded (FFPE) tissue blocks were stored at RT until use.

### Opal immunofluorescence staining

FFPE placental villous and decidual tissue samples were cut into 4-μm-thin sections on a microtome (HM 355S, Thermo Fisher Scientific). The sections were incubated in an oven for 1 h at 65 °C, deparaffinised with xylene, rehydrated with decreasing graded ethanol solutions and fixed in 10% neutral buffered formalin for 20 min. Heat-mediated antigen retrieval was performed in antigen retrieval pH 6.0 buffer (AR6, AR600250ML, PerkinElmer), followed by quenching the endogenous peroxidase activity by incubating the tissue sections in 3% hydrogen peroxide (H2O2) (107,209; Merck, Darmstadt, Germany) for 15 min at RT. The sections were then blocked with blocking/antibody diluent (ARD1001EA, PerkinElmer) for 10 min at RT. Next, the tissue sections were incubated with a mouse anti-CD62P primary antibody (1:100, ab6632, Abcam) for 1 h at RT. The slides were washed (3 × 5 min) in PBS with Tween 20 (PBST) and incubated with Opal polymer HRP Ms + Rb secondary antibody (ARH1001EA, PerkinElmer) for 10 min at RT. After being washed with PBST, the slides were incubated with Opal 540 (1:70, FP1494001 KT, PerkinElmer) for 10 min at RT. Heat-mediated antibody stripping was then performed for 15 min in the AR6 solution. This was followed by staining with the second primary antibody (rabbit anti-CD42b antibody, 1:200, ab183345, Abcam) for 3 h at RT, and the entire procedure was repeated from blocking to incubation with Opal 650 (1:100, FP1496001 KT, PerkinElmer) and antibody stripping in the AR6 solution. Finally, Spectral DAPI (4′,6-diamidino-2-phenylindole) reagent (FP1490 A, PerkinElmer) was applied to the slides for 10 min at RT, after which they were washed and mounted with ProLong TM Diamond Antifade Mountant (P36961, Thermo Fisher Scientific).

### Image analysis

A Mantra System (Mantra 1.0.2, PerkinElmer) was used to acquire images. Image processing, including unmixing fluorophores and autofluorescence, was performed using the inForm software (inForm 2.4.1, PerkinElmer). Moreover, haematoxylin and eosin (H&E)-like views of the fluorescence images were generated using the inForm program for integrated analysis. Images were analysed using ImageJ software (version 1.54f: National Institute of Health, USA). The target signals in the intervillous (maternal) areas of the placental villous tissue and decidua were quantified. Regions of interest (ROIs) were chosen using an automatic thresholding tool. The integrated density of the ROI was measured and recorded. Furthermore, colocalization analysis was performed using Just Another Colocalization Plugin (JACoP) in ImageJ software [49, 50]. We evaluated the colocalization of CD42b and CD62P fluorescence signals using Pearson’s coefficient (PCC) and Manders’ coefficient (MC). The PCC measures the relationship between the signal intensity of two markers [49, 51–53], whereas the MC measures the percentage of one molecule (i.e., CD42b) that colocalises with a second molecule (i.e., CD62P) independent of signal proportionality [51, 52, 54, 55]. A total of 5 to 10 images were quantified for each sample. Data from all these images were then transferred to Microsoft Excel and compiled to yield average values for each sample.

### Platelet isolation

Platelets were isolated from whole blood by differential centrifugation according to a previously published protocol [56]. Before centrifugation, the blood tubes were inverted six times to increase the platelet yield. The whole blood tube was centrifuged at 120 × *g* for 20 min at RT. Approximately ≤ 9/10 of the platelet-rich plasma was then collected and centrifuged at 360 × *g* for 20 min at RT to pellet the platelets. Leukocyte depletion was performed using CD45^+^ magnetic beads according to the manufacturer’s recommendations (Miltenyi Biotec, Bergisch Gladbach, Germany). Briefly, the platelet pellet was gently resuspended in buffer (PBS, 0.5% bovine serum albumin and 2 mM EDTA), and CD45 MicroBeads were added. The mixture was incubated for 15 min at RT and washed with buffer. Magnetic separation columns (130,042,201, Miltenyi Biotec) were placed in the magnetic field of a magnetic activated cell sorting (MACS) separator and loaded with a platelet suspension to remove CD45^+^ cells by negative selection. The purified platelets were subsequently obtained by centrifugation at 500 × *g* for 10 min at RT.

Platelets were also isolated from the tissue samples (i.e., placental villous and decidual tissues) using a previously described method [57, 58], which was modified for the cell type of interest. After the tissues were thoroughly washed with cold sterile PBS, the pieces were transferred into appropriately labelled 50-ml tubes. The tissue pellet was then resuspended in accutase cell detachment solution (07922, Stem cell technologies). The homogenised tissues were transferred to C-tubes (130,093,237, Miltenyi Biotec), which were placed in a gentleMACS™ dissociator (130,093,235, Miltenyi Biotec) to digest the tissues mechanically. The tissues were then incubated with accutase cell detachment solution for 80 min at 37 °C with gentle agitation. Following incubation, PBS was added to the digestive mixture to inactivate the enzyme. The digestion mixture was then passed through 100- and 40-μm cell strainers into new 50-ml tubes. The 50-ml tubes were filled with PBS and centrifuged at 300 × *g* for 5 min at RT to remove the supernatant. A human platelet isolation kit (ab289839, Abcam) was used to isolate platelets from the cell suspension. Accordingly, the resulting cell pellet was loaded on top of the 5-ml density barrier solution in a 15-ml tube and centrifuged at 350 × *g* for 15 min at RT. The layer of density barrier solution containing platelets was transferred to a new 15-ml tube, diluted with equal volumes of platelet storage buffer, and centrifuged at 200 × *g* for 10 min at RT. The supernatant was removed, and leukocyte depletion was performed as described above. Each step of centrifugation was supplemented with PGI2 (P6188, Sigma Aldrich) to prevent exogenous platelet activation due to sample processing. The platelet purity was assessed by complete cell count using a Sysmex XN-350 automated analyser. Following isolation, the platelet pellets were reconstituted in 700 µl of QIAzol Lysis Reagent (Qiagen GmbH, Hilden, Germany). The suspension was then vortexed for 1 min and promptly stored at − 80 °C before RNA extraction.

### Library preparation and RNA-seq

Total RNA was extracted from the platelets using miRNeasy Mini Kit (Qiagen, Cat. 217,004) according to the manufacturer’s instructions. Following extraction, the RNA was immediately stored at − 80 °C. The concentration and integrity of the RNA were assessed using NanoDrop ND-2000 spectrophotometer (Thermo Fisher Scientific, Waltham, MA, USA) and Agilent 2100 bioanalyzer (Agilent Technologies, Palo Alto, CA, US). RNA-seq was performed by BGI, Hong Kong. Briefly, mRNAs and lncRNAs were enriched by removing ribosomal RNA (rRNA) from the total RNA. A random hexamer-primed reverse transcription process was used to synthesise first-strand cDNA from the fragmented RNAs. This was followed by second-strand cDNA synthesis with dUTP. End repair and single-nucleotide A (adenine) addition were performed after cDNA fragment purification. The cDNA fragments were subsequently inked with adaptors, and the second strand was subsequently digested with uracil-N-glycosylase (UNG). A suitably sized cDNA fragment was chosen for PCR amplification. Finally, the cDNA libraries were converted into DNA nanoballs for paired-end 100 bp (PE100) sequencing on the DNBSEQ platform.

### RNA-seq data processing and analysis

Before downstream bioinformatics analyses, reads with low quality (> 50% low-quality bases in a read), adapter contamination, high levels of unknown N bases (> 10% N bases), and duplicate reads were filtered using SOAPnuke v1.5.2 [59] to ensure the reliability of the results. The remaining clean reads were mapped to the human reference genome GRCh38 using the map software HISAT2 v2.0.4 [60]. The reads were then assembled with StringTie v1.0.4 [61] and processed using Cufflinks v2.2.1 [62]. The expression of genes was calculated using RNA-Seq by Expectation Maximization (RSEM) software package [63].

### Differential gene expression analysis

Differential gene expression analysis was performed using the DESeq2 software package [64]. DEGs were identified based on a *q* value threshold of ≤ 0.05. DEG analysis was performed separately for the high-risk preeclampsia (HR-PE) vs high-risk without preeclampsia (HR-nPE) groups and the HR-nPE vs low-risk without preeclampsia (LR-nPE) groups. The overlapping genes identified in both the HR-PE vs HR-nPE and HR-nPE vs LR-nPE comparisons were excluded from the downstream analyses to minimise the potential confounding effects of aspirin.

We subsequently performed functional enrichment analyses of the differentially expressed mRNAs using the Dr. Tom program (BGI). These analyses included Gene Ontology (GO) functional terms and the Kyoto Encyclopedia of Genes and Genomes (KEGG) signalling pathways. To reduce redundancy, related GO terms were clustered and visualised using a web-based portal, Metascape. The significance of enrichment was set at a *q* value ≤ 0.05.

### Gene set enrichment analysis

To identify the gene sets associated with preeclampsia, GSEA was performed using previously published gene sets of activating and inhibiting pathways in platelets [34, 65]. This analysis included all the genes. We first calculated an enrichment score, which indicates the degree of overrepresentation of a gene set at either the top or bottom of the ranked list of genes. Using the gene set permutation test, we estimated the statistical significance (nominal *p* value) of the enrichment score. A normalised enrichment score (NES) was used to compare the analysis results across gene sets. This is because NES considers the size difference between gene sets and their correlation with expression data [66]). To prevent inaccurate normalisation of enrichment scores, gene sets containing fewer than 15 genes or more than 500 genes were filtered out of the analysis.

### Validation of RNA-seq results

Candidate protein-coding genes with consistent differential expression across the preeclamptic cases compared to nonpreeclamptic controls were selected for quantitative real-time polymerase chain reaction (RT‒qPCR) validation. Moreover, we selected genes whose expression was specific to each sample type and involved in significantly enriched KEGG signalling pathways. Peripheral blood, placental villous and decidual tissue samples were collected from other women to validate the DEGs identified through RNA-seq. Platelet isolation and RNA extraction were then performed according to the methods described above.

A complementary strand of DNA (cDNA) was generated from total RNA using random hexamers (N8080127, Invitrogen), SuperScript IV Reverse Transcriptase Kit (18,090,050, Invitrogen) and deoxynucleoside triphosphates (dNTP) Mix (18,427,013, Thermo Scientific). The concentration was adjusted to an equivalent of 10 ng/µl using RT‒PCR-grade water (AM9935, Invitrogen). The template RNA was combined with random hexamers and dNTP mix, briefly centrifuged, and incubated at 65 °C for 5 min, followed by a 1-min cooling period on ice. The reverse transcription reaction mix was subsequently prepared by combining 5xSSV (superScript IV reverse transcription) buffer, dithiothreitol (DTT), RNase inhibitor (10,777,019, Invitrogen) and SuperScript IV reverse transcriptase. The reverse transcription mix was then added to the annealed RNA, and the combined reaction mix was loaded onto the ProFlex PCR System (Applied Biosystems, USA) with the following thermal cycling program: 23 °C for 10 min, 55 °C for 10 min and 80 °C for 10 min.

RT‒qPCR was performed using a LightCycler 480 instrument (Roche Diagnostics, Mannheim, Germany) with a 5-µl PCR reaction mix. The reaction mix consisted of TaqMan Universal Master Mix II, no UNG (4,440,040, Applied Biosystems), target-specific primers along with probes (Thermo Fisher Scientific), cDNA, and RT‒PCR grade water. Each sample was run in triplicate. The primers used in this study are shown in Additional file 2: Table S1. Glyceraldehyde-3-phosphate dehydrogenase (GAPDH) was used as the reference gene for normalisation. The delta‒delta Ct (ΔΔCt) method was used to determine the relative expression of each gene. The delta Ct (ΔCt) values were initially calculated by subtracting the Ct value of GAPDH from the Ct value of the target gene for each sample. The ΔCt values were further calibrated using the average ΔCt value of the nPE control group: ΔΔCt = (Ct_target gene_ − Ct_GAPDH_) PE − (Ct_target gene_ − Ct_GAPDH_) nPE. The results were then presented as 2^−ΔΔCt^.

### Statistical analysis

The normal distribution of the data was evaluated using the Kolmogorov‒Smirnov test. Continuous data are presented as median (interquartile range), whereas categorical data are reported as frequency (percentage). For continuous data, the Mann‒Whitney *U* test and the Kruskal‒Wallis test were used to compare differences between two groups and multiple groups, respectively. The differences between groups were compared by the Wilcoxon signed-rank test for paired samples. Fisher’s exact test or the chi-square test was used to compare categorical variables.

The levels of the platelet indices were expressed as multiple of the median (MoM), which was derived by dividing the observed values by the expected values of the platelet indices at a particular gestational age. The expected values of PC, MPV and PC/MPV ratio were determined based on the data of the aspirin-naïve nonpreeclamptic (ASA-naive nPE) group. Moreover, platelet indices at each time point were converted to their log_10_ values to allow for a normal distribution of the measurements before statistical analysis. The repeated measures data were then analysed using a linear mixed effects model, which handles missing data without requiring imputation. The fixed effect components included height, weight at booking, maternal age, body mass index (BMI) at booking, parity, chronic hypertension, diabetes mellitus, smoking, mode of conception, aspirin compliance, gestational age at platelet indices measurement, study groups and interaction between gestational age at platelet indices measurement and study groups. The intercept (subject identification) and gestational age at platelet indices measurement were included in the random-effect component. The best model was determined using the likelihood ratio (LRT) test (the random intercept or random slope model, the intercept-slope model and the base model with no random effects were compared). All pairwise comparisons of platelet indices between groups and within-group changes over time were adjusted for multiple tests using Sidak’s method [67]. The results are presented as estimated marginal means and 95% CIs. Logistic regression analysis was also performed to determine the associations between platelet indices and aspirin nonresponsiveness. The multivariate logistic regression model included maternal factors that were significantly different between the aspirin-treated preeclamptic (ASA-PE) and aspirin-treated nonpreeclamptic (ASA-nPE) groups. Receiver operating characteristic (ROC) analysis was then performed to evaluate the discriminatory ability of the logistic regression model. Finally, the area under the curve (AUC) values of the regression models were compared using the DeLong test [68].

Statistical analysis was performed using SPSS 26.0 software (IBM, Armonk, New York, USA), MedCalc for Windows (version 22.017; Ostend, Belgium) and GraphPad Prism for Windows (version 8.0.2; San Diego, CA, USA). A value of *p* < 0.05 was considered statistically significant.

## Results

### Sociodemographic characteristics

During the study period of the longitudinal analysis of platelet indices, 489 and 1674 women at low and high risk for preeclampsia, respectively, gave birth to a live baby. We excluded 167 women due to gestational hypertension. A total of 993 women with platelet indices data were included in this study. Among these, 109, 658 and 226 women were included in the ASA-PE, ASA-nPE and ASA-naïve nPE groups, respectively (Additional file 2: Fig. S1). In the aspirin-treated groups, there was higher proportion of parous women with previous history of preeclampsia, chronic hypertension and diabetes mellitus. Furthermore, they had higher BMI (Table 1).

**Table 1:** Demographic and clinical characteristics of the study participants in longitudinal study

Characteristics	ASA-PE (*n* = 109)	ASA-nPE (*n* = 658)	ASA-naïve nPE (*n* = 226)	*p* value
Age (years)	34.3 (30.3–36.8)	34.6 (31.6–38.1)	34.2 (31.9–36.7)	0.149
Weight at booking (kg)	63.5 (55.6–73.5)	59.3 (52.6–68.3)	55.3 (51.9–61.2)	** < 0.001**
Height (cm)	160 (156–164)	159 (155–163)	160 (156–163)	0.085
BMI at booking (kg/m^2^)	25.4 (22.0–27.9)	23.5 (20.9–27.1)	21.9 (20.2–23.9)	** < 0.001**
Smoking				
Yes	10 (9.2)	57 (8.7)	9 (4)	0.06
No	99 (90.8)	601 (91.3)	217 (96)	
Family history of PE				
Yes	3 (2.8)	9 (1.4)	1 (0.4)	0.195
No	106 (97.2)	649 (98.6)	225 (99.6)	
Parity				
Nulliparous	77 (70.6)	453 (68.8)	137 (60.6)	** < 0.001**
Parous, no prior PE	18 (16.6)	166 (25.3)	85 (37.6)	
Parous, prior PE	14 (12.8)	39 (5.9)	4 (1.8)	
Chronic hypertension				
Yes	22 (20.2)	30 (4.6)	0 (0)	** < 0.001**
No	87 (79.8)	628 (95.4)	226 (100)	
Diabetes mellitus				
Yes	5 (4.6)	25 (3.8)	1 (0.4)	**0.028**
No	104 (95.4%)	633 (96.2)	225 (99.6)	
Mode of conception				
Spontaneous	102 (93.6)	603 (91.6)	209 (92.5)	0.758
In vitro fertilisation	7 (6.4)	55 (8.4)	17 (7.5)	
ASA compliance	98.1 (91.5–100)	99.3 (96.2–100)	NA	0.051
GA at PE diagnosis (weeks)	36 (34.3–37.2)	NA	NA	NA
PE subtype				
Preterm PE	48 (44)	NA	NA	NA
Term PE	61 (56)	NA	NA	NA
GA at delivery (weeks)	36.9 (35.1–37.3)	38.3 (37.6–39.1)	38.9 (38.1–39.9)	** < 0.001**
Mode of delivery				
Vaginal	25 (22.9)	355 (54)	150 (66.4)	** < 0.001**
Caesarean section	84 (77.1)	303 (46)	76 (33.6)	
Birth weight (kg)	2.3 (1.8–2.8)	2.9 (2.7–3.2)	3.1 (2.9–3.4)	** < 0.001**

Data are presented as median (25 th percentile–75 th percentile) for continuous variables and *n* (%) for categorical variables. *ASA *aspirin, *BMI *body mass index, *GA *gestational age, *NA *not applicable, *nPE *nonpreeclampsia, *PE *preeclampsia. Significant values are in bold

Opal immunofluorescence staining was performed on 83 placental villous and 48 decidual tissue samples. Compared with the other groups, the ASA-PE group had a higher BMI, delivered babies with a lower birth weight, and had a lower gestational age at delivery (Additional file 2: Table S2).

Platelet RNA-seq was also performed on 40 samples from 5 HR-PE, 5 HR-nPE and 6 LR-nPE women. There was no significant difference in maternal demographics across the three groups. However, HR-PE women delivered a lower birth weight baby and had a lower gestational age at delivery (Additional file 2: Table S3). RT‒qPCR analysis was performed on samples from 6 HR-PE and 6 HR-nPE women to validate the RNA-seq results. Compared with the HR-nPE group, the HR-PE group had a lower gestational age at delivery. In addition, a longer time interval between aspirin stoppage and delivery of a baby was observed in the HR-nPE group (Additional file 2: Table S4).

### Longitudinal changes in platelet indices

The random intercept model for PC and the random intercept‒random slope model for MPV and PC/MPV ratio provided a better fit to the data. The best-fit linear mixed effect model demonstrated that increasing maternal height and weight were associated with lower and higher PC, respectively (*p* < 0.001). Moreover, there was a significant effect of gestational age and the interaction between the groups and gestational age on PC, MPV and PC/MPV ratio (Additional file 2: Table S5).

**Fig. 1:**
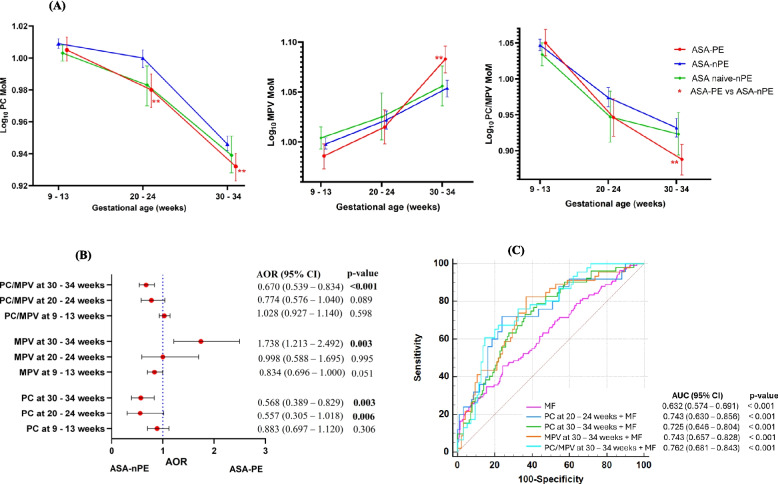
Longitudinal evaluation of platelet indices. **A** Comparison of platelet indices across gestation. **B** Logistic regression analysis for platelet indices at different gestation periods, adjusted for maternal factors (BMI, chronic hypertension, and parity). **C** ROC analysis of platelet indices in combination with maternal factors. PC: platelet count, MOM: multiple of the median, MPV: mean platelet volume, ASA: aspirin, PE: preeclampsia, nPE: nonpreeclampsia, AOR: adjusted odds ratio, CI: confidence interval, MF: maternal factors, AUC: area under the curve. **p < 0.01

PC was highest in the ASA-nPE group, followed by the ASA-naïve nPE and ASA-PE groups. Overall comparisons between the ASA-PE and ASA-nPE groups, as well as between the ASA-nPE and ASA-naïve nPE groups, showed significant differences in PC (*p* = 0.005 and *p* = 0.035, respectively). Pairwise comparisons showed that PC was significantly lower in the ASA-PE group than in the ASA-nPE group at 20–24 (*p* = 0.003) and 30–34 weeks (*p* = 0.008). A lower PC/MPV ratio was also observed in the ASA-PE group than in the ASA-nPE group at 30–34 weeks (*p* = 0.002). In all the groups, PC and PC/MPV ratio tended to decrease across gestation. In contrast, the MPV was significantly higher in the ASA-PE group than in the ASA-nPE group at 30–34 weeks of gestation (*p* = 0.001). Longitudinal changes in MPV in all groups continuously increased across gestation (Additional file 2: Table S6, Fig. 1A).

### Associations of platelet indices with aspirin nonresponsiveness

A multivariate logistic regression analysis (adjusted for BMI, chronic hypertension and parity) showed that PC at 20–24 weeks and PC, MPV and PC/MPV at 30–34 weeks of gestation were significantly associated with aspirin nonresponsiveness (Fig. 1B). ROC analysis revealed that platelet indices alone had a modest discriminatory ability for aspirin nonresponsiveness, with AUCs between 0.627 and 0.684 (Additional file 2: Table S7). However, the multivariate model improved the AUC to 0.743, 0.725, 0.743 and 0.762 for PC at 20–24 weeks and PC, MPV and PC/MPV at 30–34 weeks of gestation, respectively. Pairwise comparisons of the regression models indicated that combining platelet indices with maternal factors yielded a significant increase in the AUC compared with that of maternal factors alone and platelet indices alone (Fig. 1C, Additional file 2: Table S7).

### Platelet activation at the maternal–foetal interface

**Fig. 2:**
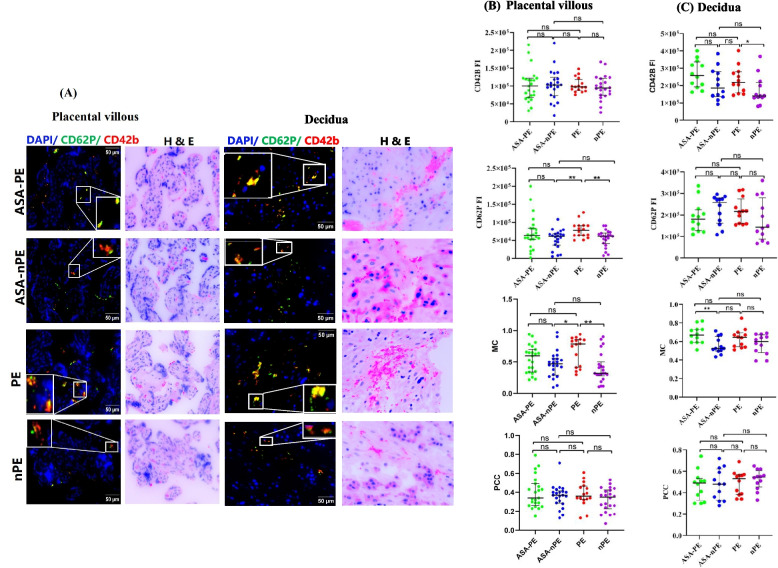
Activation of platelets in placental villous and decidual tissues. **A** Representative Opal immunofluorescence staining images with individual and merged channels (magnification: × 400, scale bar: 50 μm). **B** Expression and colocalization of CD42B and CD62P in placental villous. **C** Expression and colocalization of CD42B and CD62P in decidual tissues. ASA: aspirin, PE: preeclampsia, nPE: nonpreeclampsia, FI: fluorescence intensity, MC: Mander’s coefficient, PCC: Pearson’s coefficient, ns: nonsignificant. *p < 0.05, **p < 0.01

Analysis of the staining intensity in the placental villous tissue showed that CD62P expression was significantly higher in the PE group than in the nPE (*p* = 0.009) and ASA-nPE (*p* = 0.007) groups. Moreover, analysis of MC showed that the PE group exhibited a high degree of colocalization between CD42b and CD62P compared to the nPE (*p* = 0.002) and ASA-nPE (*p* = 0.022) groups (Fig. 2A, B).

In decidual tissue, CD42b expression in the PE group was significantly higher compared to the nPE group (*p* = 0.02). The ASA-PE decidua had a significantly higher MC than did the ASA-nPE decidua (*p* = 0.008, Fig. 2A,C).

The expression of CD42b and CD62P was significantly higher in decidua than in placental villous tissues in all study groups (*p* < 0.01). Furthermore, the ASA-PE and ASA-nPE decidua had significantly higher MC than the ASA-PE and ASA-nPE placental villous, respectively (*p* < 0.01, Additional file 2: Fig. S2).

**Fig. 3:**
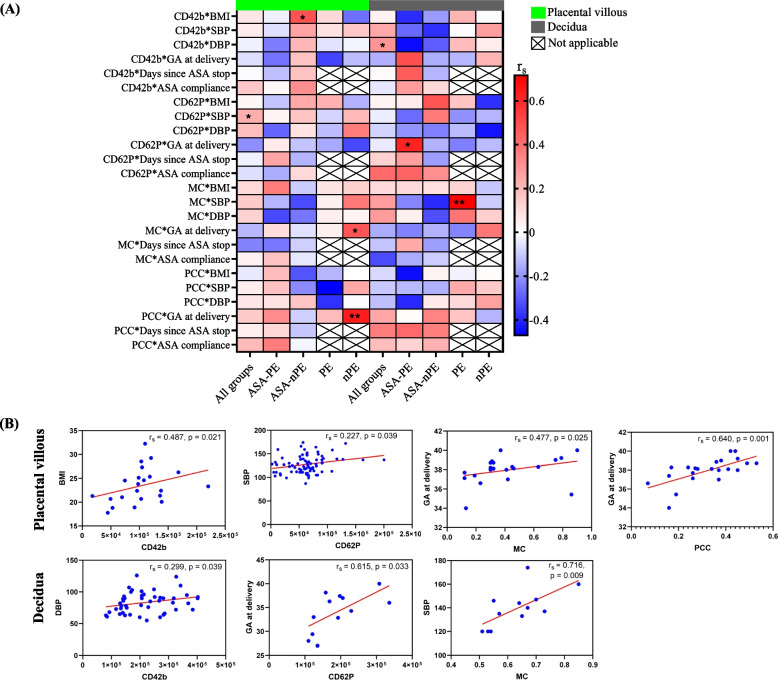
Correlations between clinical factors and activated platelets in placental villous and decidual tissues. **A** Correlation heatmap. Activated platelets and clinical factor correlations are denoted on the left side of the heatmap. The colours represent Spearman’s correlation coefficient, with intense colours indicating stronger correlations. BMI: body mass index, SBP: systolic blood pressure, DBP: diastolic blood pressure, GA: gestational age, MC: Manders’ coefficient, PCC: Pearson’s coefficient, ASA: aspirin, PE: preeclampsia, nPE: nonpreeclampsia, rs: Spearman’s correlation coefficient. *p < 0.05, **p < 0.01. **B** A scatter plot of significant correlations

According to correlation analyses based on all groups, BMI, gestational age at delivery, aspirin compliance, and time (in days) since aspirin cessation did not significantly correlate with CD42b, MC or PCC (Fig. 3A). However, there was a significant positive correlation between systolic blood pressure and CD62P expression in placental villous tissue. In addition, systolic blood pressure was positively correlated with MC in the decidua of the PE group (Fig. 3A, B).

### Differentially expressed genes identified in the RNA-seq analysis

**Fig. 4:**
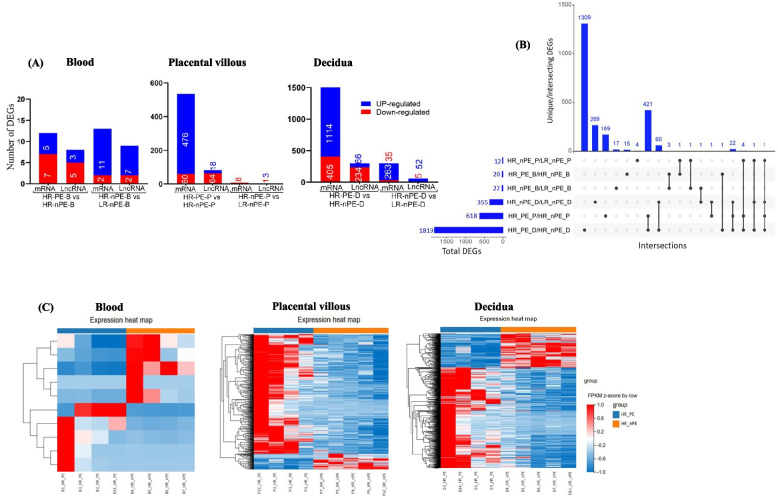
Differentially expressed genes (DEGs) in platelets. **A** Number of significantly upregulated and downregulated DEGs in the blood, placental villous and decidua. The blue and red colours indicate up- and downregulated DEGs with *q* value ≤ 0.05, respectively. **B** DEGs and overlapping in different comparison groups. The horizontal bars to the left represent the total number of DEGs in each comparison. The vertical bars represent the number of intersecting DEGs for the comparisons indicated below. DEGs shared between comparisons are indicated by dark dots in each category with connecting lines between them. The dark dots without connecting lines represent unique DEGs. **C** Hierarchical clustering map of differentially expressed mRNAs in the blood, placental villous and decidua between HR-PE and HR-nPE groups Columns indicate samples, rows indicate genes and colour intensity indicates the *Z*-score transformed gene expression values on a blue (low) to red (high) scale. HR: high risk, PE: preeclampsia, B: blood, nPE: nonpreeclampsia, LR: low risk, P: placental villous, D: decidua

Each sample generated an average of 9.07 Gb of data, with 4.4% of the raw reads filtered (2.6–7.2%). The average alignment ratio of the sample comparison genome was 96.74% (range 91.98–97.75%), with 80.74% of reads per sample mapped uniquely. The quality metrics and alignment results for the RNA-seq data are presented in Additional file 2: Table S8. Differential gene expression analysis between HR-PE and HR-nPE identified 20, 618 and 1819 DEGs in the blood, placental villous and decidua, respectively (Additional file 3, Fig. 4A, B). Most of the DEGs were upregulated in preeclampsia. Moreover, a comparison of HR-nPE with LR-nPE revealed 22, 12 and 355 DEGs in the blood, placental villous and decidua, respectively (Fig. 4A, B). Hierarchical clustering of the differentially expressed mRNAs revealed differences in the gene expression profiles of the HR-PE and HR-nPE groups (Fig. 4C).

### Functional analyses of the differentially expressed mRNAs

**Fig. 5:**
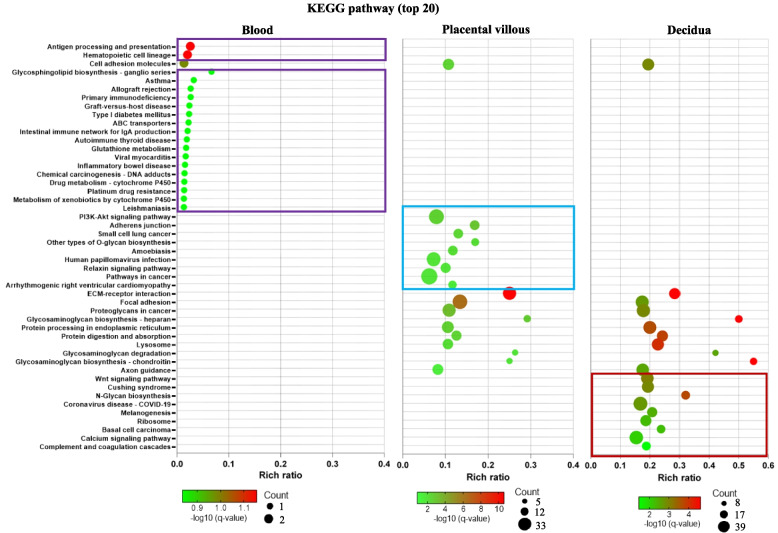
Kyoto Encyclopedia of Genes and Genomes (KEGG) analysis of differentially expressed mRNAs in platelets obtained from blood, placental villous and decidua in the HR-PE vs HR-nPE groups. The colours of the circles and boxes represent the *q* value. The size of the circle represents the gene count

Functional enrichment analyses were conducted to gain further insight into the biological functions of the differentially expressed mRNAs. In the peripheral blood, GO enrichment analysis showed that the DEGs were enriched mainly in GO terms related to chemokine signalling (Additional file 2: Fig. S3). However, the KEGG pathway enrichment analysis did not reach statistical significance (*q* > 0.05, Fig. 5).

**Fig. 6:**
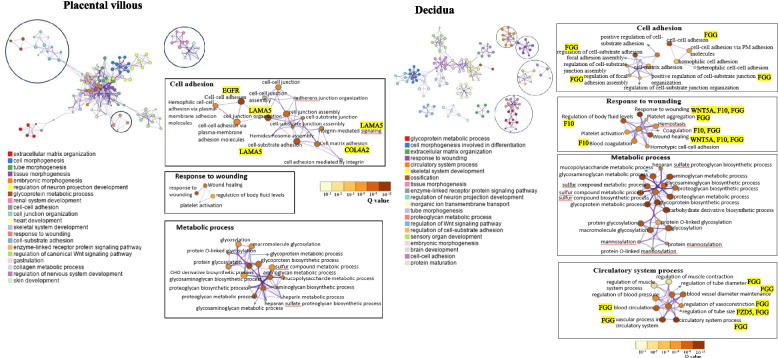
Clusters of enriched GO terms for differentially expressed mRNAs in platelets across placental villous and decidua in the HR-PE vs HR-nPE groups. The circle node and colour in the cluster network represent the GO term and cluster identity, respectively. The colour of the subnetwork represents the *q* value. The term descriptions are shown as labels for each cluster. Genes selected for RT‒qPCR validation are also linked to GO terms in the subnetwork

KEGG pathway analysis showed that the PI3 K-Akt signalling pathway was specifically enriched in the placenta (Fig. 5). The most enriched GO terms were extracellular matrix organisation, heparin-binding and collagen-containing extracellular matrix under biological process, molecular function and cellular component, respectively (Additional file 2: Fig. S3). GO cluster analysis of the placental villous revealed that the DEGs were highly clustered in biological processes associated with cell adhesion, response to wounding and metabolic process (Fig. 6).

In the decidua, the Wnt signalling pathway and complement and coagulation cascades were specifically enriched (Fig. 5). GO terms related to extracellular matrix organisation, extracellular matrix structural constituent and integral component of the membrane were significantly enriched in the biological process, molecular function and cellular component categories, respectively (Additional file 2: Fig. S3). GO cluster analysis revealed that the DEGs were highly clustered in biological processes associated with cell adhesion, response to wounding, circulatory system and metabolic processes (Fig. 6).

### Gene set enrichment analysis results

After filtering, 23, 24 and 24 gene sets were analysed in the blood, placental villous and decidua, respectively. The list of gene sets and enrichment results are shown in Additional file 2: Tables S9 and S10. The KEGG calcium signalling pathway and REACTOME integrin cell surface interactions were significantly enriched in platelets obtained from the blood, placental villous and decidua of the HR-PE group. REACTOME platelet aggregation plug formation was enriched in the decidua of the HR-PE group. However, none of the gene sets were significantly enriched in the HR-nPE group (Additional file 2: Fig. S4, Table S9 and S10).

### Quantitative real-time polymerase chain reaction results

**Fig. 7:**
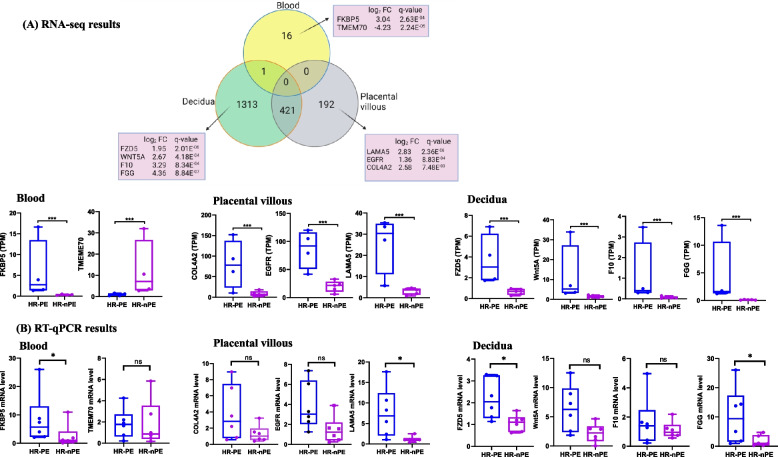
Validation of platelet DEGs in blood, placental villous and decidua. **A** Venn diagram and box plot of DEGs selected from RNA-seq data for RT‒qPCR validation. **B** RT‒qPCR validation of selected DEGs. FKBP5: FKBP prolyl isomerase 5, TMEM70: Transmembrane protein 7, FZD5: frizzled class receptor 5, WNT5 A: Wingless (Wnt) family member 5 A, F10: Coagulation factor X, FGG: Fibrinogen gamma chain, LAMA5: Laminin subunit alpha 5, EGFR: Epidermal growth factor receptor, COL4 A2: Collagen type IV alpha 2 chain, HR: high risk, PE: preeclampsia, nPE: nonpreeclampsia. *p < 0.05, ***p < 0.001

RT‒qPCR analysis revealed significant increases in FKBP5, LAMA5, FZD5 and FGG mRNA expression levels in the HR-PE group compared with those in the HR-nPE group. Furthermore, COL4 A2, EGFR, WNT5 A and F10 mRNAs were expressed at higher levels in the HR-PE group. However, statistical significance was not achieved, which might be due to the small sample size. Overall, these RT‒qPCR results were consistent with the findings obtained from RNA-seq (Fig. 7).

## Discussion

### Main findings

In this study, we evaluated the changes in platelet indices, activation state and transcriptional landscape during preeclampsia. The main findings include (1) the ASA-PE group had a lower PC and PC/MPV ratio, as well as a higher MPV compared to the ASA-nPE group in the third trimester; (2) PC at 20–24 weeks and PC, MPV and PC/MPV at 30–34 weeks of gestation were significantly associated with the subsequent development of aspirin nonresponsiveness; (3) we observed a higher degree of platelet activation in both the placental villous and decidua of women with preeclampsia than in those without preeclampsia; (4) the expression levels of CD62P and CD42b were higher in the decidua compared to the placental villous in all study groups; (5) the PI3 K-Akt and Wnt signalling pathways were significantly enriched in the placental villous and decidua of preeclamptic patients, respectively; (6) increased expression of FKBP5, LAMA5, FZD5 and FGG in platelets from preeclamptic women was confirmed by RT‒qPCR.

### Interpretation and comparison with the literature

The finding of lower PC in women who develop preeclampsia despite aspirin treatment is consistent with our earlier study [21] and other studies [18, 20, 69, 70]. However, these studies were conducted on women who had not taken aspirin during pregnancy. The persistent decrease in PC in ASA-PE women suggests that there may be a suboptimal platelet response to aspirin. In line with this, Theilen et al. demonstrated that women with a consistent increase in platelet activation (as reflected by the increase in plasma PF4 levels) during pregnancy despite aspirin treatment were at greater risk for developing hypertensive disorders of pregnancy [16]. It has been shown that platelet activation amplifies platelet consumption and clearance, decreasing PC [71–74]. The response of platelets to aspirin treatment may vary depending on several factors, including the dose of aspirin, the platelet activation pathway and the platelet turnover rate [16, 75]. For example, COX-1-independent platelet activation pathways, such as ADP-, collagen- or thrombin-induced activation, are unaffected by aspirin. Moreover, newly generated platelets in settings of increased platelet turnover tend to be more thrombogenic and less responsive to aspirin [76, 77]. Although increased platelet turnover stimulates platelet production in the bone marrow, this production may not fully compensate for increased platelet consumption, reducing PC [78–80]. Furthermore, we observed increased PC in ASA-nPE women compared with ASA-naïve nPE women. These findings suggest that there may be an increase in platelet activation and clearance in nonpreeclamptic pregnancies that can be inhibited by aspirin. Indeed, our results in the ASA-naïve nPE group agree with the known changes in PC in normal pregnancy, with a gradual decline in PC throughout gestation [81–84]. The MPV in the ASA-PE group was higher than that in the ASA-nPE group, providing further evidence that preeclamptic women may have higher platelet activation despite aspirin treatment. A higher MPV has been reported to be associated with increased platelet aggregation activity, elevated expression of platelet adhesion molecules and increased synthesis of thromboxane A2 [85–88]. The MPV increases under conditions involving higher platelet destruction and decreases under conditions of low platelet production [89]. Our findings of a lower PC and higher MPV may indicate overdestruction rather than underproduction of platelets. Furthermore, the MPV in both the ASA-nPE and ASA-naïve nPE groups progressively increased from the first trimester onwards. A similar trend was reported in other studies on normal pregnancies [90–93], indicating increased levels of platelet activation, which can be further exaggerated during preeclampsia [94, 95]. It has been suggested that the ratio of PC to the MPV may better indicate platelet activity than PC or MPV alone [86, 96, 97]. In line with our results, previous studies on aspirin-naïve pregnant women reported similar findings of a low PC/MPV ratio in a preeclamptic group compared with normotensive pregnant women in the third trimester. These studies have demonstrated that the PC/MPV ratio can be useful in assessing the risk of preeclampsia [70, 98, 99]. In our study, the moderate discriminatory power of platelet indices suggested that they could assist in identifying low-dose aspirin nonresponders.

The sequestration of maternal platelets in the intervillous area of the placenta and their adhesion to the villous surface have been suggested as common processes during human pregnancy [38, 41, 42]. Thus, platelets may play an important role in placental pathologies but have been undermined [17, 100]. A previous study indicated that the sequestration of maternal platelets in the placenta contributes to the changes in platelet indices, including a decrease in PC in the circulation during normal pregnancy [38]. Considering the placental origin of preeclampsia [3, 4] and the increased sequestration of maternal platelets in the placenta during pregnancy [38], we further investigated the potential platelet changes at the maternal-foetal interface. In the present study, higher expression of CD42b, a platelet-specific marker on the surface of both resting and activated platelets [101, 102], was detected in the preeclamptic decidua than in the nonpreeclamptic decidua, indicating the presence of abundant maternal platelets in the preeclamptic decidua. This may contribute to the increase in certain platelet-derived factors and thromboinflammation at the maternal–foetal interface since excessive platelets are associated with an increased risk of thrombotic complications [103, 104]. Moreover, the abundance of platelet staining in the decidua may also partly contribute to the lower PC observed in the systemic circulation of preeclamptic women.

Our study demonstrated that preeclampsia is associated with increased platelet activation in the placental villous and decidua, as evidenced by increased colocalization of CD42b with CD62P, an activation marker present on the surface of activated platelets [105, 106]. Consistent with this, we found that the MC was positively correlated with systolic blood pressure in preeclamptic decidua. In line with our findings, Kohli et al. reported that activated maternal platelets that accumulate in the placenta are linked to thromboinflammation and preeclampsia [40]. Although the mechanisms that initiate platelet activation in preeclampsia are unclear, they can be activated by various factors, such as trophoblast-derived extracellular vesicles and platelet-derived agonists [6, 107, 108]. Platelets release biologically active molecules from their granules upon activation, further enhancing platelet activation via autocrine and paracrine mechanisms [109]. Furthermore, in the present study, comparing CD42b and CD62P expression in the placental villous and decidua based on aspirin status revealed no significant differences between the groups. Low-dose aspirin inhibits platelet activation by inhibiting the COX-1-mediated pathway, but it does not affect other mechanisms of platelet activation [16, 110, 111]. For example, damage to syncytiotrophoblasts and exposure to extracellular matrix components, including collagen, may partly contribute to platelet activation at the maternal–foetal interface [80]. However, further studies are needed to verify our results.

The present study conducted further RNA-seq analysis to explore transcriptional dysregulation in platelets during preeclampsia. We identified a small number of DEGs in the peripheral blood between preeclamptic and normotensive women, indicating modest changes in platelet gene expression. However, we found hundreds and thousands of DEGs in the placental villous and decidua, respectively, indicating an apparent difference between the groups. Nearly half of these genes were distinct from those reported in previous platelet RNA-seq studies of other diseases, including sepsis [34, 112], COVID-19 [37, 113], human immunodeficiency virus (HIV) [114], cardiovascular diseases [11, 32], cancer [36, 115] and diabetes mellitus [116]. These findings suggest that preeclampsia is associated with unique changes in platelet gene expression. In addition, numerous genes have been identified in decidua that are unique from those found in the placental villous, suggesting that there can be tissue-specific platelet gene signatures.

Our functional enrichment analysis of the DEGs showed significant enrichment of PI3 K-Akt signalling in platelets obtained from the placental villous. This may be responsible for the increased platelet activation in the placenta [117–119]. We also identified that biological processes involved in platelet metabolism, adhesion, activation and thrombosis were enriched. Activation of PI3 K-Akt signalling pathway is believed to play an important role in mediating platelet activation, granule secretion, integrin activation and thrombosis. It has been shown that platelet-matrix interaction and shear stress-induced platelet activation involved the PI3 K/Akt signalling pathway [117–119]. The increased activation of the PI3 K-Akt signalling pathway may be partly due to increased COL4 A2, LAMA5 and EGFR expression. These genes are known upstream regulators of the PI3 K-Akt signalling pathway [120–122]. Further support for the increased activation of platelets in the preeclamptic placenta is provided by the GSEA results, which revealed a significant enrichment of activatory platelet signalling networks, including integrin cell surface interactions and calcium signalling. Integrin signalling is partly mediated by the PI3 K-Akt pathway [65, 120].

In decidua, functional enrichment analysis showed enriched terms associated with platelet metabolism, blood pressure, platelet adhesion and activation during preeclampsia. These changes are manifestations of increased platelet activation [123]. We also identified significantly enriched GO terms related to platelet aggregation. Increased FGG expression may have contributed to our observation of increased platelet aggregation. Furthermore, we observed an increase in the Wnt signalling pathway in platelets. The increased activation of the Wnt signalling pathway may be due to increased expression of WNT5 A and FZD5, which are known upstream regulators of the noncanonical (β-catenin-independent) Wnt signalling pathway. This pathway may also contribute to our observation of increased calcium signalling in platelets during preeclampsia, as Wnt-Ca^2+^ signalling enhances calcium release, stimulating Ca^2+^-dependent signalling molecules [124, 125]. Previous studies have demonstrated that both canonical, which functions through β-catenin, and noncanonical (β-catenin-independent) Wnt signalling pathways are present in platelets [126–128]. Unlike the noncanonical pathway, the Wnt-β-catenin signalling pathway inhibits the activity of platelets, including platelet activation, aggregation, exocytosis of dense granules and changes in platelet shape [126]. In RNA-seq, we did not detect significant changes in the mRNA expression of β-catenin (CTNNB1 [catenin β1]) or glycogen synthase kinase 3 beta (GSK3β), two critical downstream effectors of the canonical Wnt pathway [126, 129], suggesting that the noncanonical Wnt pathway may play an important role in platelet function during preeclampsia. The GSEA results showing increased platelet aggregation and calcium signalling further support the observations that platelet activation is increased in preeclamptic decidua.

In the peripheral blood, the KEGG analysis did not reveal any significantly enriched pathways, possibly due to the small number of genes included in this analysis. However, we observed increased expression of FKBP5 in preeclamptic women. FKBP5 has been shown to trigger a proinflammatory immune response by interacting with NF-κB signalling [130, 131]. Moreover, it has been suggested that the inhibition of FKBP immunophilins, such as FKBP5, reduces platelet procoagulant responses, including phosphatidylserine externalisation [132].This suggests that the upregulation of FKBP5 may increase platelet activity. In line with this finding, the GSEA results showed a significant enrichment of pathways related to platelet activation in women with preeclampsia. The enrichment of activatory rather than inhibitory platelet signalling networks reflects increased activation of platelets in preeclampsia [72, 133–136].

### Strengths and limitations

Our study has the following strengths: (1) we measured platelet indices at multiple time points, which allowed us to demonstrate platelet reactivity throughout gestation; (2) our results are based on a well-defined cohort with complete information on maternal medical history, demographics and clinical characteristics; (3) both preeclampsia and aspirin status were considered when investigating changes in platelets during preeclampsia. Additionally, other concomitant conditions have been excluded or adjusted in analyses to minimise the impact of potential confounders; (4) we examined the gene expression profiles of platelets obtained from different sample types for the first time in preeclampsia; (5) we collected samples from a separate group of women for RT‒qPCR validation. This approach is more convincing and reliable than performing RNA-seq and validation on the same samples. However, our study has the following limitations that should be considered when interpreting the results: (1) in this study, aspirin nonresponsiveness was defined as the development of preeclampsia despite taking aspirin. This does not explicitly reflect the inhibition of platelet COX by aspirin. However, there is no consensus regarding the definition of aspirin nonresponsiveness. It has been defined clinically, biochemically or in combination [29, 137]. A test targeting the COX pathway should be considered to determine the platelet response; (2) records with missing data on platelet indices were found in all study groups. As a result, the sample size was limited, especially from the second trimester onwards; (3) data on platelet indices were obtained retrospectively; (4) our sample size for RNA-seq and validation experiments was small, and a larger cohort is needed to verify the results; (5) the changes in platelets at the maternal‒foetal interface during delivery may not reflect their condition throughout gestation; (6) we cannot determine whether the observed transcriptomic and activation changes represent a response or play an active role in causing the disease. This is an endpoint study, and thus, cause‒effect relationships cannot be established; (7) the influence of sample preparation and the lengthy procedure of isolating platelets from the tissue on gene expression detection cannot be excluded. Currently, there is no standardised protocol to isolate platelets for RNA-seq experiments. However, all the samples were processed in the same way; (8) our analyses of platelet indices and gene expression did not include aspirin-naïve preeclamptic women. It presented a challenge in obtaining this group of women. Aspirin prophylaxis is now recommended for all pregnant women at increased risk of preeclampsia unless there are known contraindications. In addition, the screen and prevent strategy for preeclampsia is highly accepted by FORECAST participants, and more than 90% of high-risk women have received aspirin prophylaxis [47]. The observed changes in platelet indices are related to aspirin nonresponsiveness. Therefore, a comprehensive analysis of the direct association between preeclampsia and platelets across the course of gestation may warrant further study, excluding the influence of aspirin use; and (9) since our study included the Hong Kong population, more diverse ancestries should be included in future studies to broaden the generalisability of our findings.

### Clinical and research implications

The changes in platelet indices despite aspirin treatment may assist in monitoring the platelet response to aspirin, leading to more beneficial modifications to preventive therapy. Furthermore, the easy accessibility of platelet indices makes it easier to incorporate them into assessment strategies for aspirin nonresponsiveness. Understanding the underlying molecular changes in platelets and their activation state during preeclampsia can also assist in identifying the causative mediators and potential therapeutic targets that play a role in the pathogenesis of preeclampsia and its adverse outcomes, such as thrombohemorrhagic complications. Moreover, many of the identified molecules may provide new insights into potential biomarkers of preeclampsia for further investigation. Our findings may open further studies evaluating the role of platelets at the maternal‒foetal interface. Future studies are still needed to evaluate the following aspects: (1) although our study demonstrated that PC, MPV and PC/MPV ratio are associated with aspirin nonresponsiveness, future large-scale prospective studies are still needed to verify our findings; (2) future studies should investigate the factors contributing to inadequate suppression of platelet activation despite aspirin treatment in women at high risk for preeclampsia. Several factors, including pharmacokinetic, pharmacodynamic and genetic factors, have been proposed to explain the insufficient inhibition of platelet function by aspirin. In addition, poor adherence, inadequate dosing and increased turnover of platelets have been implicated [31, 111, 138, 139]. Such studies among women at high risk for preeclampsia will be important to maximise the positive effects of aspirin; (3) the maternal platelet cargo, their interaction with other cells and the consequences of platelet activation at the maternal–foetal interface should be investigated; (4) further functional studies should be considered for a deeper understanding of the relationship between platelets and preeclampsia pathogenesis. This may help identify novel drug alternatives to aspirin for preventing, delaying and managing preeclampsia.

## Conclusions

We found that PC in the second trimester and PC, MPV and PC/MPV ratio in the third trimester may be useful for assessing aspirin nonresponsiveness in women at high risk of preeclampsia. In addition, women with preeclampsia demonstrated an increase in platelet activation at the maternal–foetal interface, as well as significant enrichment of signalling pathways involved in platelet activation. Our findings could drive further investigations targeting the interconnection between preeclampsia pathophysiology and platelet activation to establish biomarkers and uncover novel prevention or treatment targets.

## Supplementary Information

Additional file 1. STROBE checklist of items in reports of observational studies.

Additional file 2. Figure S1. Participant selection flowchart. Figure S2. The expression and colocalization of CD42b and CD62P in placental villous and decidual tissues. FI: fluorescence intensity, ASA: aspirin, PE: preeclampsia, nPE: nonpreeclampsia, MC: Mander’s coefficient, PCC: Pearson’s coefficient, **p < 0.01, ns: nonsignificant. Figure S3. Gene Ontology (GO) analysis of differentially expressed mRNAs in platelets obtained from blood, placental villous and decidua in the HR-PE vs HR-nPE groups. The top 15 enriched GO terms in the biological process (BP), cellular component (CC), and molecular function (MF) categories. The colour and size of the circle represent the q value and gene count, respectively. Figure S4. Enrichment plots in blood, placental villous and decidua using GSEA. NES: normalised enrichment score, HR: high risk, PE: preeclampsia, nPE: nonpreeclampsia. Table S1. Primers used in the RT‒qPCR experiment. Table S2. Demographic and clinical characteristics of the study participants in Opal immunofluorescence staining. Table S3. Demographic and clinical characteristics of the study participants in RNA-seq. Table S4. Demographic and clinical characteristics of the study participants in the RT‒qPCR validation. Table S5. Linear mixed-effects models for PC, MPV and PC/MPV: fixed effects. Table S6. Pairwise comparisons of platelet indices across gestation. Table S7. Screening performance of platelet indices and maternal factors for aspirin nonresponsiveness. Table S8. The quality control metrics and alignment results for the RNA-seq data. Table S9. Gene sets upregulated in the high-risk aspirin-treated with preeclampsia (HR-PE) group. Table S10. Gene sets upregulated in the high-risk aspirin-treated without preeclampsia (HR-nPE) group

Additional file 3. Lists of differentially expressed genes between HR-PE and HR-nPE groups.

## Data Availability

The datasets used and/or analysed during the current study are available from the corresponding author on reasonable request.

## Abbreviations

ASA: Aspirin
DEG: Differentially expressed genes
HR: High risk
LR: Low risk
MC: Mander’s coefficient
MoM: Multiple of the median
MPV: Mean platelet volume
nPE: Nonpreeclamptic
PC: Platelet count
PCC: Pearson’s correlation
PE: Preeclamptic
RT: Room temperature
