# Supplementary material for: Platelets in preeclampsia: an observational study of indices associated with aspirin nonresponsiveness, activation and transcriptional landscape
Source: BMC Med. 2025 Jun 9;23:346. doi: 10.1186/s12916-025-04132-9 (PMC12150514; doi:10.1186/s12916-025-04132-9)
Supplement: Supplementary file 2 — Additional file 2. Figure S1. Participant selection flowchart. Figure S2. The expression and colocalization of CD42b and CD62P in placental villous and decidual tissues. FI: fluorescence intensity, ASA: aspirin, PE: preeclampsia, nPE: nonpreeclampsia, MC: Mander’s coefficient, PCC: Pearson’s coefficient, **p < 0.01, ns: nonsignificant. Figure S3. Gene Ontology (GO) analysis of differentially expressed mRNAs in platelets obtained from blood, placental villous and decidua in the HR-PE vs HR-nPE groups. The top 15 enriched GO terms in the biological process (BP), cellular component (CC), and molecular function (MF) categories. The colour and size of the circle represent the q value and gene count, respectively. Figure S4. Enrichment plots in blood, placental villous and decidua using GSEA. NES: normalised enrichment score, HR: high risk, PE: preeclampsia, nPE: nonpreeclampsia. Table S1. Primers used in the RT‒qPCR experiment. Table S2. Demographic and clinical characteristics of the study participants in Opal immunofluorescence staining. Table S3. Demographic and clinical characteristics of the study participants in RNA-seq. Table S4. Demographic and clinical characteristics of the study participants in the RT‒qPCR validation. Table S5. Linear mixed-effects models for PC, MPV and PC/MPV: fixed effects. Table S6. Pairwise comparisons of platelet indices across gestation. Table S7. Screening performance of platelet indices and maternal factors for aspirin nonresponsiveness. Table S8. The quality control metrics and alignment results for the RNA-seq data. Table S9. Gene sets upregulated in the high-risk aspirin-treated with preeclampsia (HR-PE) group. Table S10. Gene sets upregulated in the high-risk aspirin-treated without preeclampsia (HR-nPE) group [file 12916_2025_4132_MOESM2_ESM.zip › Table S4.pdf]

Characteristics	HR-PE (n = 6)	HR-nPE (n = 6)	p-value
Age (years)	37 (32.8 – 39.2)	35.5 (29.0 – 39.5)	0.699
Weight at booking (kg)	69.9 (53.7 – 86.0)	59.4 (54.9 – 68.4)	0.631
Height (cm)	160 (153 – 166)	158 (151 – 165)	0.687
BMI at booking (kg/m^2)	27.3 (22.4 – 31.1)	23.9 (22.0 – 28.9)	0.589
Systolic blood pressure (mmHg)	148.5 (132.3 – 165.5)	117 (109.5 – 126)	**0.037**
Diastolic blood pressure (mmHg)	89 (79.8 – 97.5)	76 (69 – 83.3)	**0.030**
Family history of PE			
Yes	0 (0)	0 (0)	NA
No	6 (100)	6 (100)	
Parity			
Nulliparous	4 (66.7)	5 (83.3)	1.000
Parous, no prior PE	2 (33.3)	1 (16.7)	
Parous, prior PE	0 (0)	0 (0)	
Mode of conception			
Spontaneous	6 (100)	6 (100)	NA
In vitro fertilisation	0 (0)	0 (0)	
ASA compliance	96.7 (84.3 – 100.0)	97.4 (93.3 – 100)	0.699
Days since ASA stop	0 (0 – 9.75)	15.5 (12.5 – 22.0)	**0.015**
GA at PE diagnosis (weeks)	32.4 (30.8 – 37.1)	NA	NA
PE subtype			
Preterm PE	4 (66.7)	NA	NA
Term PE	2 (33.3)	NA	
GA at delivery (weeks)	32.9 (31.2 – 37.4)	38.2 (37.8 – 38.9)	**0.009**
Mode of delivery			
Vaginal	1 (16.7)	2 (33.3)	1.00
Caesarean section	5 (83.3)	4 (66.7)	
Birth weight (kg)	1.5 (1.3 – 3.2)	2.8 (2.7 – 2.9)	0.394

Data are presented as median (25th percentile – 75th percentile) for continuous variables and n (%) for categorical variables. HR: high risk, PE: preeclampsia, nPE: non-preeclampsia, BMI: body mass index, NA: not applicable, ASA: aspirin, GA: gestational age. Significant values are in bold.
