# Supplementary material for: Platelets in preeclampsia: an observational study of indices associated with aspirin nonresponsiveness, activation and transcriptional landscape
Source: BMC Med. 2025 Jun 9;23:346. doi: 10.1186/s12916-025-04132-9 (PMC12150514; doi:10.1186/s12916-025-04132-9)
Supplement: Supplementary file 2 — Additional file 2. Figure S1. Participant selection flowchart. Figure S2. The expression and colocalization of CD42b and CD62P in placental villous and decidual tissues. FI: fluorescence intensity, ASA: aspirin, PE: preeclampsia, nPE: nonpreeclampsia, MC: Mander’s coefficient, PCC: Pearson’s coefficient, **p < 0.01, ns: nonsignificant. Figure S3. Gene Ontology (GO) analysis of differentially expressed mRNAs in platelets obtained from blood, placental villous and decidua in the HR-PE vs HR-nPE groups. The top 15 enriched GO terms in the biological process (BP), cellular component (CC), and molecular function (MF) categories. The colour and size of the circle represent the q value and gene count, respectively. Figure S4. Enrichment plots in blood, placental villous and decidua using GSEA. NES: normalised enrichment score, HR: high risk, PE: preeclampsia, nPE: nonpreeclampsia. Table S1. Primers used in the RT‒qPCR experiment. Table S2. Demographic and clinical characteristics of the study participants in Opal immunofluorescence staining. Table S3. Demographic and clinical characteristics of the study participants in RNA-seq. Table S4. Demographic and clinical characteristics of the study participants in the RT‒qPCR validation. Table S5. Linear mixed-effects models for PC, MPV and PC/MPV: fixed effects. Table S6. Pairwise comparisons of platelet indices across gestation. Table S7. Screening performance of platelet indices and maternal factors for aspirin nonresponsiveness. Table S8. The quality control metrics and alignment results for the RNA-seq data. Table S9. Gene sets upregulated in the high-risk aspirin-treated with preeclampsia (HR-PE) group. Table S10. Gene sets upregulated in the high-risk aspirin-treated without preeclampsia (HR-nPE) group [file 12916_2025_4132_MOESM2_ESM.zip › Table S9.pdf]

No.	Pathway	Original gene size	Blood			Placental villous			Decidua		
			Gene size	NES	NOM p value	Gene size	NES	p value	Gene size	NES	NOM p value
1	REACTOME G alpha 12 13 signalling	80	71	1.7	**0.007**	76	0.59	0.996			
2	REACTOME G alpha I signalling	314	204	1.86	**<0.001**	252	0.69	1.000			
3	REACTOME G alpha Q signalling	217	147	1.21	0.098				190	1.06	0.282
4	REACTOME G alpha Z signalling	48	36	0.8	0.802	43	1.08	0.306	43	1.33	0.083
5	REACTOME integrin cell surface interactions	85	69	1.68	**0.010**	81	2.29	**<0.001**	85	2.14	**<0.001**
6	REACTOME integrin signalling	27	24	1.15	0.229	25	0.59	0.976	27	1.18	0.226
7	REACTOME RAP1 signalling	16	15	0.75	0.799						
8	REACTOME platelet aggregation plug formation	39	32	1.09	0.316	36	1.21	0.186	38	1.82	**<0.001**
9	REACTOME thromboxane signalling through TP receptor	24	20	1.43	0.067						
10	REACTOME ADP signalling through P2Y purinoceptor 1	25	21	1.41	0.078						
11	REACTOME ADP signalling through P2Y purinoceptor 12	22	17	0.98	0.480						
12	REACTOME PLC beta mediated events	42	37	0.81	0.783	39	1.14	0.262	38	1.14	0.266
13	BIOCARTA RAC1 pathway	21	20	1.51	0.061	20	0.48	1.000	20	0.65	0.937
14	BIOCARTA RHO pathway	21	20	1.76	**0.013**	20	0.59	0.97	20	0.57	0.961
15	BIOCARTA PAR1 pathway	19	17	0.97	0.460	18	0.59	0.961			
16	BIOCARTA integrin pathway	34	32	1.29	0.128				34	0.69	0.931
17	BIOCARTA SPPA pathway	16									
18	BIOCARTA PTDINS pathway	23	23	1.17	0.248	23	0.8	0.735			
19	BIOCARTA PKC pathway	7									
20	BIOCARTA PLC pathway	9									
21	BIOCARTA EPHA4 pathway	8									
22	KEGG phosphatidylinositol signalling	76	67	1.31	0.069						
23	KEGG calcium signalling pathway	178	132	1.59	**<0.001**	155	1.5	**<0.001**	159	1.56	**0.002**
24	REACTOME PKA activation in glucagon signalling	17				15	1.86	**<0.001**	15	1.71	**0.01**
25	REACTOME prostacyclin signalling through IP	19	15	1.3	0.156						
26	REACTOME adenylate cyclase activating pathway	10									
27	BIOCARTA GPCR pathway	30	28	1.28	0.168	29	0.71	0.892			
28	BIOCARTA NOS1 pathway	20	17	1.11	0.305	18	0.6	0.952			

The empty spaces represent gene sets that are not involved. NES: normalized enrichment score, Nom: nominal.
