# Supplementary material for: Platelets in preeclampsia: an observational study of indices associated with aspirin nonresponsiveness, activation and transcriptional landscape
Source: BMC Med. 2025 Jun 9;23:346. doi: 10.1186/s12916-025-04132-9 (PMC12150514; doi:10.1186/s12916-025-04132-9)
Supplement: Supplementary file 2 — Additional file 2. Figure S1. Participant selection flowchart. Figure S2. The expression and colocalization of CD42b and CD62P in placental villous and decidual tissues. FI: fluorescence intensity, ASA: aspirin, PE: preeclampsia, nPE: nonpreeclampsia, MC: Mander’s coefficient, PCC: Pearson’s coefficient, **p < 0.01, ns: nonsignificant. Figure S3. Gene Ontology (GO) analysis of differentially expressed mRNAs in platelets obtained from blood, placental villous and decidua in the HR-PE vs HR-nPE groups. The top 15 enriched GO terms in the biological process (BP), cellular component (CC), and molecular function (MF) categories. The colour and size of the circle represent the q value and gene count, respectively. Figure S4. Enrichment plots in blood, placental villous and decidua using GSEA. NES: normalised enrichment score, HR: high risk, PE: preeclampsia, nPE: nonpreeclampsia. Table S1. Primers used in the RT‒qPCR experiment. Table S2. Demographic and clinical characteristics of the study participants in Opal immunofluorescence staining. Table S3. Demographic and clinical characteristics of the study participants in RNA-seq. Table S4. Demographic and clinical characteristics of the study participants in the RT‒qPCR validation. Table S5. Linear mixed-effects models for PC, MPV and PC/MPV: fixed effects. Table S6. Pairwise comparisons of platelet indices across gestation. Table S7. Screening performance of platelet indices and maternal factors for aspirin nonresponsiveness. Table S8. The quality control metrics and alignment results for the RNA-seq data. Table S9. Gene sets upregulated in the high-risk aspirin-treated with preeclampsia (HR-PE) group. Table S10. Gene sets upregulated in the high-risk aspirin-treated without preeclampsia (HR-nPE) group [file 12916_2025_4132_MOESM2_ESM.zip › Table S10.pdf]

No.	Pathway	Original gene size	Blood			Placental villous			Decidua		
			Gene size	NES	NOM p value	Gene size	NES	Nom p value	Gene size	NES	Nom p value
1	REACTOME G alpha 12 13 signalling	80							77	-0.8	0.862
2	REACTOME G alpha I signalling	314							267	-1	0.445
3	REACTOME G alpha Q signalling	217				176	-0.68	1			
4	REACTOME G alpha Z signalling	48									
5	REACTOME integrin cell surface interactions	85									
6	REACTOME integrin signalling	27									
7	REACTOME RAP1 signalling	16				15	-1.34	0.108	15	-0.95	0.509
8	REACTOME platelet aggregation plug formation	39									
9	REACTOME thromboxane signalling through TP receptor	24				22	-1.25	0.167	22	-0.97	0.508
10	REACTOME ADP signalling through P2Y purinoceptor 1	25				23	-1.2	0.206	23	-1	0.457
11	REACTOME ADP signalling through P2Y purinoceptor 12	22				19	-1.28	0.153	19	-1.17	0.254
12	REACTOME PLC beta mediated events	42									
13	BIOCARTA RAC1 pathway	21									
14	BIOCARTA RHO pathway	21									
15	BIOCARTA PAR1 pathway	19							18	-0.78	0.769
16	BIOCARTA integrin pathway	34				33	-0.66	0.945			
17	BIOCARTA SPPA pathway	16	15	-0.67	0.884	15	-0.67	0.894	15	-1.09	0.342
18	BIOCARTA PTDINS pathway	23							23	-0.52	0.992
19	BIOCARTA PKC pathway	7									
20	BIOCARTA PLC pathway	9									
21	BIOCARTA EPHA4 pathway	8									
22	KEGG phosphatidylinositol signalling	76				72	-0.71	0.951	73	-0.45	1
23	KEGG calcium signalling pathway	178									
24	REACTOME PKA activation in glucagon signalling	17									
25	REACTOME prostacyclin signalling through IP	19				17	-1.21	0.227	17	-0.8	0.719
26	REACTOME adenylate cyclase activating pathway	10									
27	BIOCARTA GPCR pathway	30							29	-0.71	0.885
28	BIOCARTA NOS1 pathway	20							18	-0.66	0.902

The empty spaces represent gene sets that are not involved. NES: normalized enrichment score, Nom: nominal. normalised enrichment score, HR: high risk, PE: preeclampsia, nPE: non-preeclampsia.
